# Notes and News

**Published:** 1893-09-16

**Authors:** 


### Notes and News.

Collected and Communicated.

Mr. Diggle, Chairman of the London School Board, is appealing through the columns of the *Times* for help towards the Children's Country Holiday Fund. People who have ample opportunities for getting away from the heat and dust of London have found this summer's unusually high temperature a trial. What, then, must it have been to the poor little lives spent in the slums of the great city? The amount now at the disposal of the committee is less by £500 than at the same period of last year. Will not all those who have enjoyed a country trip themselves do something towards giving the children also a breath of fresh air before the summer is quite gone? All gifts should be sent to the Hon. Treasurer, Children's Country Holiday Fund, 10, Buckingham Street, Strand, W.C.

What an excellent work the London Young Women's Christian Association is doing! A work, too, that is so much needed amongst the working girls of our City—servants, barmaids, clerks, workroom and factory girls, as the most taking little circular appeals which has reached us, sets forth. What a charming picture of cheerfulness and comfort the reading-room at the Central Institute presents. For the more actively inclined the gymnasium affords employment, whilst for those inclined to improve themselves there are all manner of classes where practically useful subjects are taught, and music not forgotten. In connection with the work there are forty-two institutes, homes, and restaurants, and numerous branches of the Society throughout the metropolis. The members' roll is now over 17,000, but we shall hope to hear of a still greater number next year, for the attractions offered to working girls, many of whom possess either miserable homes or none at all, are likely to prove of the utmost service, and the civilising tendency of such a mission can hardly be over-estimated. We advise our readers to send to the Hon. Emily Kinnaird, 115, Mount Street, W., for an account of this good work, which we feel sure only requires to be better known to meet with a hearty response to the appeal for assistance both to extend and maintain the undertaking.

The Chancellor of the Duchy of Lancaster has placed Dr. A. Envoys-Jones, of Manchester, on the commission of the peace for that city.

The Victoria Hospital for Children, Queen's Road, Chelsea, which has been closed for repairs, is now again ready for the reception of patients. The building has been fitted throughout with the electric light. The Committee ask all friends of the hospital to pay a visit of inspection any afternoon between two and five o'clock, an invitation which should be largely responded to.

One of the principal difficulties, if they understand their responsibility rightly, which the authorities of orphanages have to incur is the placing of children on leaving the homes. This is illustrated by the result of an appeal made by the secretary of a well-known orphanage to the well-to-do to adopt orphans. Here is the reply: "Mrs. —— has decided on adopting two healthy female orphans, age about 17. One must be able to do plain cooking and scrubbing, the other to do general housework and wait at table. Both must be early risers, sober, and industrious."

We are glad to notice that members of the medical profession in the provinces are calling attention to articles in The Hospital. A recent instance of this is the discussion that has been carried on in a Monmouthshire paper on the subject of cycling and heart disease, based on an annotation in our columns thus entitled. If our article acts as a warning to the many really immoderate cyclists we shall feel we have done some good, for a really excellent form of recreation becomes imperilled by the indiscreet to the disadvantage of the many who are wiser.

The foundation-stone of the Charnwood Forest Convalescent Home was laid on the 2nd ult. by the Duchess of Rutland, a large company being present. The Home is to serve for the whole of Leicestershire, and is to accommodate fifty patients. The movement has been one of gradual growth, the outcome of necessity. Existing buildings in different localities have been abandoned one after another, as affording insufficient room, and eventually a fund for a new and adequate building was started. That fund now amounts to £4,547, and the estimated cost of the Home is £6,000. No doubt as to the subscription of the deficit is entertained, and eventually it is hoped to provide a children's wing.

There is an old story of some man who studied the subject of the adulteration of various popular articles of diet until he grew so impressed by the dangers attendant on food that he gradually gave up one thing after the other, eventually dying of want of nourishment. We may hope to avoid his fate, but still our indulgences are being daily restricted. We have to submit to boiled milk, which is no great hardship, and to boiled water, which is certainly nasty, and yet we know it to be wholesome, and so we dare not rebel. In their zeal against harmful things, some people would prohibit good as well as bad fruit, entirely forgetting that in food as well as drink temperance means moderation, not necessarily "total abstinence." But the latest dictum that we must not clean our teeth with ordinary filtered water unless it is also boiled is surely converting common-sense precautions into the similitude of absurdities.

